# Effectiveness of tranexamic acid in reducing blood loss in spinal surgery: a meta-analysis

**DOI:** 10.1186/1471-2474-15-448

**Published:** 2014-12-22

**Authors:** Fan Zhang, Kun Wang, Feng-Ning Li, Xuan Huang, Quan Li, Zhi Chen, Yi-Bo Tang, Hong-Xing Shen, Qing-Xin Song

**Affiliations:** Department of Spine Surgery, Changhai Hospital, the Second Military Medical University, No. 168 Changhai Road, Yangpu District, Shanghai 200433 China; Department of Orthopedics, Shanghai Eastern Hepatobiliary Surgery Hospital, Second Military Medical University, Shanghai, China

**Keywords:** Tranexamic acid, Spine, Surgery, Meta-analysis

## Abstract

**Background:**

The aim of present meta-analysis was to evaluate the effectiveness of tranexamic acid (TXA) use in reducing blood loss and the related thrombotic complications in spinal surgery.

**Methods:**

Three databases (MEDLINE, EMBASE, and the Cochrane Library) were searched through October 2012 to identify the relevant randomized controlled trials (RCTs) regarding the TXA effective in spinal surgery. Mean differences (MDs) of blood loss, blood transfusions, and postoperative partial thromboplastic time (PTT), odds ratios (ORs) of blood transfusion and thrombotic complication in TXA-treated group compared to placebo group were extracted and combined using random-effect meta-analysis.

**Results:**

A total of 6 RCTs comprising 411 patients were included in the meta-analysis according to the pre-defined selection criteria. TXA-treated group had significantly less amount of blood loss and blood transfusions per patient, and had smaller proportion of patients who required a blood transfusion compared with the placebo group. The use of TXA can significantly reduce the postoperative PTT with weighted MD of -1.59 [(95% confidence interval (CI):-3.07, -0.10] There is a null association between thrombosis complications and the use of TXA.

**Conclusion:**

We conclude that the use of TXA in patients undergoing spinal surgery appears to be effective in reducing the amount of blood loss, the volume of blood transfusion, the transfusion rate, and the postoperative PTT. However, data were too limited for any conclusions regarding safety. More high-quality RCTs are required before recommending the administered of TXA in spinal surgery.

**Electronic supplementary material:**

The online version of this article (doi:10.1186/1471-2474-15-448) contains supplementary material, which is available to authorized users.

## Background

Given the complexity of spinal surgeries, bleeding will be inevitably faced during spinal surgery. Many patients have to receive allogeneic blood transfusion because of excessive blood loss, which may result in immunologic reactions or transmitting infections, even cause transfusion-related acute lung injury [1, 2]. Another concern with regard to perioperative bleeding in spinal surgery is the risk of spinal epidural hematoma formation, which might lead to spinal cord or cauda equina compression [3]. Therefore, reducing blood loss both intra- and postoperatively presents a challenge to the spine surgeon [4, 5].

Various strategies, such as controlled hypotensive anesthesia, the cell salvage system, fresh-frozen plasma and cryoprecipitate, to reduce the intraoperative blood loss during spinal surgery have been attempted. Tranexamic acid (TXA) is a synthetic antifibrinolytic drug that competitively blocks the lysine binding sites of plasminogen, plasmin, and tissue plasminogen activator, thereby retarding fibrinolysis and blood clot degradation. It can decrease intra- and postoperative bleeding by acting on the fibrinolytic system theoretically [6]. It has been confirmed that TXA use could play a role in reducing blood loss in cardiac surgery and hip or knee arthroplasty [7, 8], However, the effectiveness of TXA used in spinal surgery was controversial in several studies [9–13]. Thus, we conducted a meta-analysis of randomized controlled trials (RCTs) to evaluate the effectiveness of TXA for the reduction of blood loss in spinal surgery.

## Methods

### Literature search

We comprehensively identified studies through searching MEDLINE (PubMed), EMBASE, and the Cochrane Library through October 2012 for all RCTs published in English regarding the effective and safe of the TXA use in spinal surgery were searched from three databases. The reference lists of retrieved articles were also manual scanned to locate additional relevant studies. The following key words were used for search: tranexamic acid, spine, spinal.

### Inclusion and exclusion criteria

All RCTs about the TXA use in the spinal surgery were performed. We systematically reviewed published studies according to the following criteria: (1) randomized controlled trials; (2) subjects were underwent spinal fusion including cervical, thoracic and lumber spine and adolescent scoliosis correction surgery irrespective of anterior or posterior approach; (3) the intervention was TXA intravenous(IV) administered an experimental group that used TXA, a control group that received a placebo, intravenous administration at the beginning of surgery in both groups, and (4) the primary outcome measures should include one of the following outcomes: amount of total blood loss, amount of allogeneic blood transfusion, ratio of blood transfusion and thrombosis complications, such as deep vein thrombosis (DVT) or pulmonary embolism.

Studies should be excluded if they: 1) were nonrandomized controlled clinical trials; 2) had a low quality; 3) they had no interventions described above; 4) had a non-intravenous administration drug; and 5) did not contain any of the above outcomes.

### Literature retrieve and quality assessment

Two investigators independently reviewed all titles, abstracts, and the full text of articles that were potentially eligible based on abstract review. Then the eligible trials were selected according to the inclusion criteria. Disagreement was resolved by discussion if necessary, or by involving a third reviewer for adjudication. We assessed the study quality using the method described by Jadad et al. [14], in which a study was judged on 3 aspects as follows: 1) random assignment (full score = 2); 2) blinding investigators and patients (full score = 2), and 3) the proportion of dropouts and withdrawals in the follow-up study (full score = 1) Thus the full was 5 points. Studies with 3 points or higher were considered to represent high-quality research and were included in the meta-analysis.

### Statistical analysis

For eligible studies, relevant data was extracted by two investigators independently, including the data about the amount of blood loss, the amount of allogeneic blood transfusion, the ratio of blood transfusion and DVT or other thromboembolism, partial thromboplastic time (PTT), thromboembolic complications, and any other outcomes as mentioned in included studies. Of 6 included RCTs [15–20], all studies reported the mean differences (MDs) between TXA and placebo group for the total blood loss and the amount of blood transfusion per patient; all studies reported the odds ratio (OR) of blood transfusion in TXA-treated group compared to placebo group (referent); four studies [15–18] reported MD for postoperative partial thromboplastic time (PTT); two studies [15, 18] reported MD for PTT at 24 h postoperative; whereas only one study [17] reported the OR of thrombotic complication for TXA. Therefore we selected OR of blood transfusion for the use of TXA, and MDs between TXA and placebo group for the total blood loss, the amount of blood transfusion per patient, postoperative PTT, and PTT at 24 h postoperative as the effects of intervention. Statistical analysis were conducted using Review Manager (RevMan) version 5.0 (The Cochrane Library, Oxford, United Kingdom). For the amount of blood loss, the amount of blood transfusion, PTT, the weighted mean differences with a 95% confidence intervals (CIs) were combined in the meta-analysis; while for the rate of patients needing transfusion, the incidence of deep-vein thrombosis, and the rate of pulmonary embolism, odds ratios (ORs) with 95% CIs were combined. All the pooled analysis used random-effect models. Heterogeneity across studies was detected using Cochran’s Q and I^2^ statistics.

## Results

### Study selection and involved studies’ characteristics

**Figure 1:**
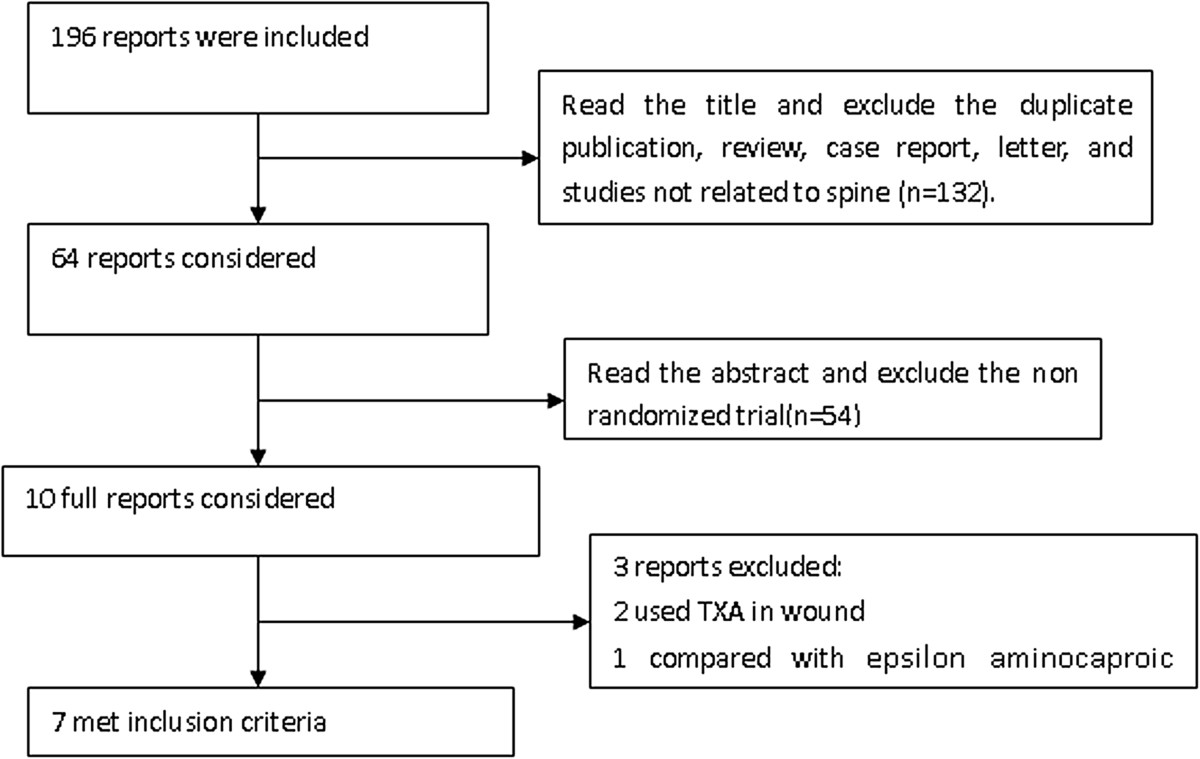
**Study selection process.** A flowchart was present in this figure and the flowchart summarizes the selection of studies including numbers and reasons of studies excluded.

Figure 1 summarizes the process for identifying eligible studies. The search strategy yielded a total of 172 citations, of which 105 were eliminated on the basis of the title review. TXA and spine were involved in 67 studies, while only 9 had a RCT design. After full text review of remaining 9 studies, one studies was excluded because TXA in this study is not via intravenous administration; one study was excluded because it used the different control method, in which epsilon aminocaproic acid was used in control group. At last, six RCTs [15–20] involving 411 patients were included in the final analysis, with individual sample sizes ranging from 40 to 147 patients (Table 1). The quality scores of the included studies were above 4 points according the Jadad’s scoring-system. The characteristics of included studies were presented in Table 2. Two studies [15, 16] is about correction surgery, in which patients aged 8 to 18 year with either primary or secondary scoliosis, one study focused on posterior approach, and another focused on both anterior and posterior approach, and part of the patients accepted the autologous bone harvest procedure. Two studies [17, 18] were about the thoracic, thoracolumbar or lumbar spinal decompression and instrumented fusion; Two studies [19, 20] were about cervical spinal surgery, one focused on laminoplasty only, and another one focused on anterior cervical discectomy and laminectomy. No significant differences in the baseline information was observed between the TXA and placebo groups, including the patient’s age, sex, height, weight, and hemoglobin. Blood transfusion were undertaken if the hemoglobin was less than 10 g/L in Farrokhi’s study [18] which was higher than the others. The TXA was given as a loading dose administered intravenously at the start of surgery in all studies, followed by maintenance dose during the surgery in 5 studies, only initiation dose of TXA was given in Tsutsumimoto’s study [20]. Different doses and time of delivery of TXA were used. Four studies used relatively lower a dosage of TXA which ranged from 10 to 15 mg/kg, whereas 2 used a relatively higher dosage of TXA (100 mg/kg or 2 g for adults, 30 mg/kg for children).

**Table 1:** **Basic information of the studies involved in the meta-analysis**

Studies	Number of pts (TXA/P)	Sex		Age		Weight	
		Male (TXA/P)	Female (TXA/P)	TXA	Placebo	TXA	Placebo
Neilipovitz DT, 2001 [15]	22/18	17 (12/5)	23 (10/13)	14.1 ± 2.1	13.7 ± 2.5	41.8 ± 16.7	50.6 ± 20.2
Sethna NF, 2005 [16]	23/21	30 (17/13)	14 (6/8)	13.6 ± 1.8	14.0 ± 2.0	59.4 ± 18.3	52.4 ± 15.7
Wong J, 2008 [17]	73/74	47 (21/26)	100 (52/48)	56.8 ± 16.2	50.0 ± 16.2	72.9 ± 17.2	73.9 ± 16.1
Elwatidy S, 2008 [19]	32/32	39 (21/18)	25 (11/14)	51.56 ± 19.08	49.75 ± 21.04	72.48 ± 13.81	69.63 ± 17.29
Farrokhi MR, 2011 [18]	38/38	18 (11/7)	58 (27/31)	45.5 ± 11.6	51.4 ± 11.6	66.6 ± 9.9	66.9 ± 9.4
Tsutsumimoto T, 2011 [20]	20/20	31 (16/15)	9 (4/5)	68.0 ± 11.0	65.8 ± 11.8	59.6 ± 8.3	61.3 ± 10.2

Pts, patients; TXA/P, tranexamic acid group/placebo group.

**Table 2:** **Characteristics of included studies**

Study	Type	Intervation	Surgery	Outcome measure	Complication	Indication of transfusion	Jadad’ score
Neilipovitz DT, 2001 [15]	RCT	I: 10 mg/kg	Posterior spinal fusion for scoliosis, 16 pts accepted autologous bone harvest	Blood loss, blood transused, Hb, Plt, PTT, DVT	0	Hb ≤ 7 g/L	5
		M: 1 mg/kg/h					
		P: Unclear					
Sethna NF, 2005 [16]	RCT	I: 100 mg/kg	Thirty six pts for posterior spinal instrumentation and 8 for anterior–posterior instrumentation	Blood loss, blood transused, Hct, PT, Plt, PTT, DVT	0	Hct ≤ 25%	5
		M: 10 mg/kg/h					
		P: Saline					
Wong J, 2008 [17]	RCT	I: 10 mg/kg	Thoracic, thoracolumbar or lumbar spinal decompression and instrumented fusion	Blood loss, blood transused, Hb, PT, PTT, DVT	TXA: 1 pts MI	Hb ≤ 7 g/L	5
		M: 1 mg/kg/h			P: 1 pts DVT		
		P: Saline					
Elwatidy S, 2008 [19]	RCT	I: 2 g for adouts, 30 mg/kg for children	Eighteen pts had multiple level anterior cervical discectomy with or without fixation, 37 had spinal decompression for multisegment spinal stenosis, 9 had laminectomy and excision of spinal tumor	Blood loss, blood transused, Hb, Hct, DVT	0	Hb ≤ 9 g/L	5
		M: 100 mg/h for adouts, 1 mg/kg/h for children				Hct ≤ 27%	
		P: Saline					
Farrokhi MR, 2011 [18]	RCT	I: 10 mg/kg	Posterior thoracic or lumbar instrumented spinal fusion at 4 to 6 vertebrae	Blood loss, blood transused, Hb, PT, PTT, DVT	0	Hb ≤ 10 g/L	5
		M: 1 mg/kg/h					
		P: Saline					
Tsutsumimoto T, 2011 [20]	RCT	I: 15 mg/kg	Cervical laminoplasty from C3 to C6	Blood loss, Hb, Hct, PT, PTT, DVT, hematoma formation	0	Not mention	4
		P: Saline					

Pts, patients; I, initiation dose of tranexamic acid; M, maintenance dose of tranexamic acid; P, placebo; Hb, hemoglobin; Plt, platelets; Hct, hematocrit; PT, prothrombin time; PTT, partial thromboplastin time; MI, myocardial infarction; DVT, deep venous thrombosis.

### Amount of blood loss

**Figure 2:**
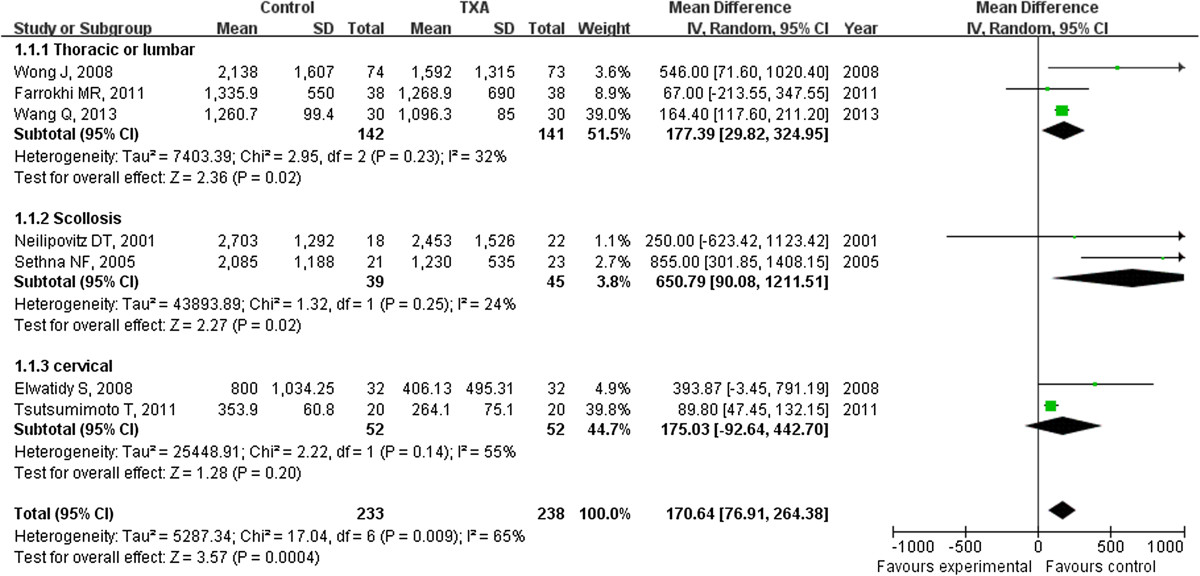
**The average amount of blood loss was significantly less in the TXA group compared with the placebo group (weighted mean difference, -100.68 ml [95% CI, -142.00 to -59.36 ml]; p < 0.00001); similar result in the scoliosis sub group (weighted mean difference, -681.81 ml [95% CI, -1149.12 to -214.49 ml]; p = 0.004) and non-scoliosis subgroup (weighted mean difference, -96.10 ml [95% CI, -137.59 to -54.62 ml]; p < 0.00001;).** TXA = tranexamic acid, SD = standard deviation, IV = inverse variance, and df = degrees of freedom.

**Figure 3:**
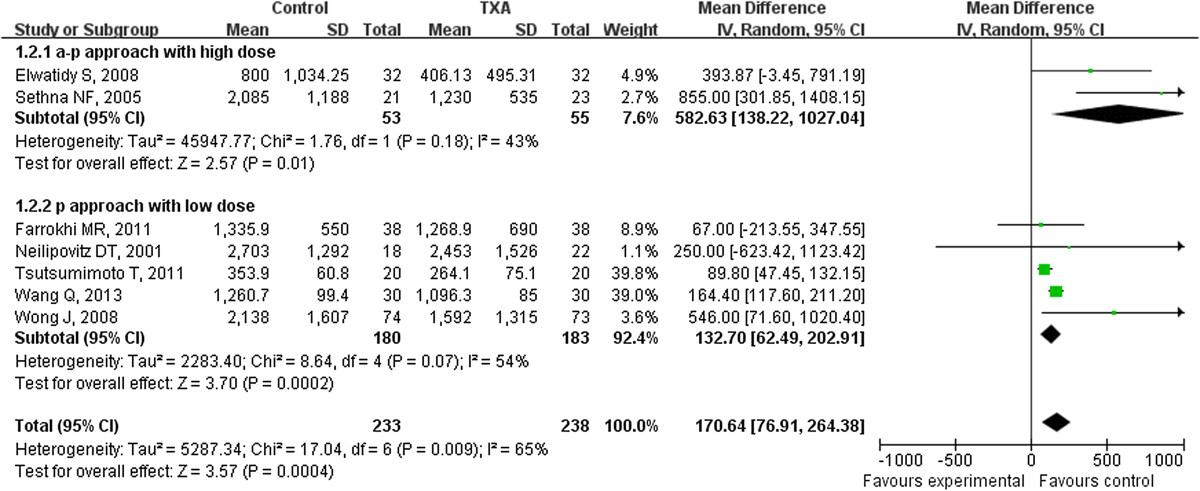
**The average amount of blood loss was significantly less in the TXA group, either in subgroup of anterior-posterior approach with high TXA dose or in subgroup of posterior approach with low TXA dose (weighted mean difference, -550.81 ml [95% CI, -873.51 to -228.11 ml]; p = 0.0008; weighted mean difference, -93.18 ml [95% CI, -134.84 to -51.52 ml]; p < 0.0001 respectively;). TXA = tranexamic acid, SD = standard deviation, IV = inverse variance, and df = degrees of freedom.**

Data on total blood loss were available in all six studies. Our analysis showed perioperative intravenous administration of TXA can significantly reduce the total blood loss with MD of -100.68 ml (95% CI: -142.00 ml to -59.36 ml) compared to placebo groups. Since the index of I^2^ is 62%, some clinical heterogeneity were considered. In order to observe the potential source of such a heterogeneity, subgroup analysis by surgical methods, surgical approach and IV TXA dosage were conducted. First, subgroup analysis was performed according to the surgical methods. The amount of blood loss per patient significantly decreased with MDs of 681.81 ml (95% CI: 214.49 to 1149.12 ml) and 96.10 ml (95% CI: 54.62 to 137.59 ml) in the TXA scoliosis subgroup and TXA non scoliosis subgroup respectively, and the value of I^2^ decreased by 24% and 48% respectively (Figure 2). Second, subgroup analysis by surgical approach and IV TXA dose were performed, the amount of blood loss per patient significantly decreased with MDs of 550.81 ml (ranging from 228.11 to 873.51 ml) and 93.18 ml (ranging from 51.52 to 134.84 ml) for the anterior-posterior approach with high dose TXA subgroup and for the posterior approach with low dose TXA subgroup respectively, and the value of I^2^ decreased by 43% and 19% respectively (Figure 3).

### Amount of blood transfusion

**Figure 4:**
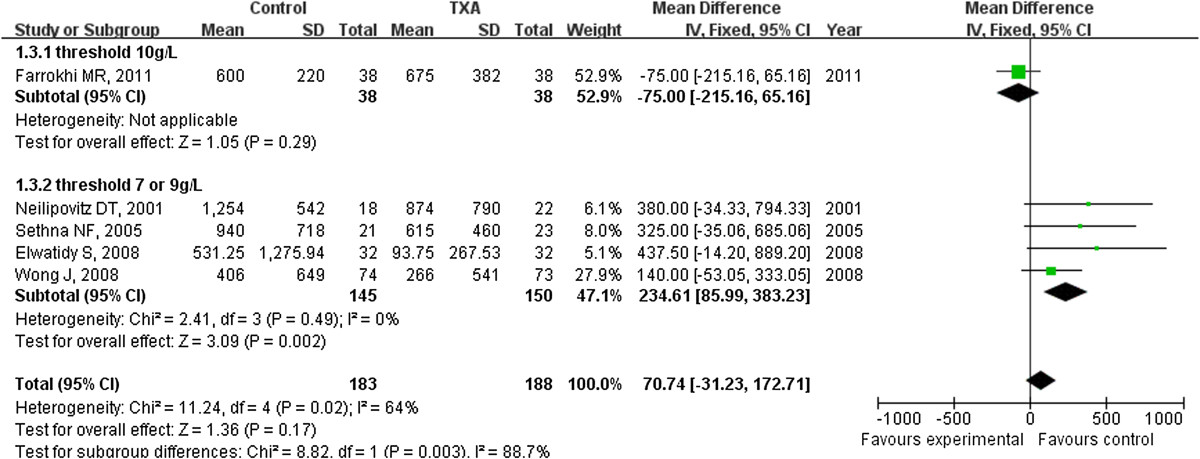
**The amount of blood transfusion was significantly less in the TXA group compared with the placebo group (weighted mean difference, -234.61 ml [95% CI, -383.23 to -85.99 ml]; p = 0.002).** TXA = tranexamic acid, SD = standard deviation, IV = inverse variance, and df = degrees of freedom.

The number of patients requiring transfusion after surgery was available in all six studies. The pooled weighted MD for the amount of blood transfusion per patient between TXA group and placebo group is -234.61 ml (95% CI: -383.23 to -85.99 ml) (Figure 4). However, there was significant heterogeneity within the selected studies.

### Odds ratio of blood transfusion

**Figure 5:**
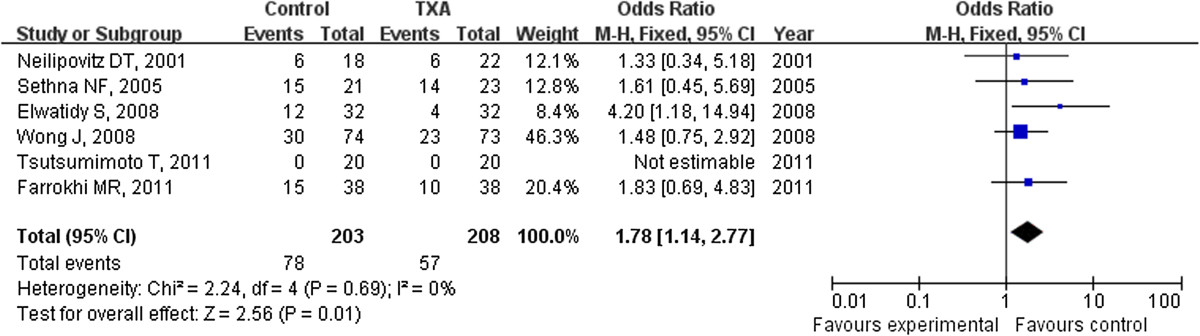
**The ratio of patients who accepted the blood transfusion was significantly less in the TXA group compared with the placebo group (weighted mean difference, 0.56 [95% CI, 0.36 to 0.87]; p = 0.01).** TXA = tranexamic acid, SD = standard deviation, IV = inverse variance, and df = degrees of freedom.

The incidence of blood transfusion were provided in all six studies. The TXA use could decreased 44% risk of blood transfusion compared with the placebo group with a OR of 0.56 (95% CI: 0.36 to 0.87) (Figure 5).

### Odds ratio of thrombotic complication

**Figure 6:**
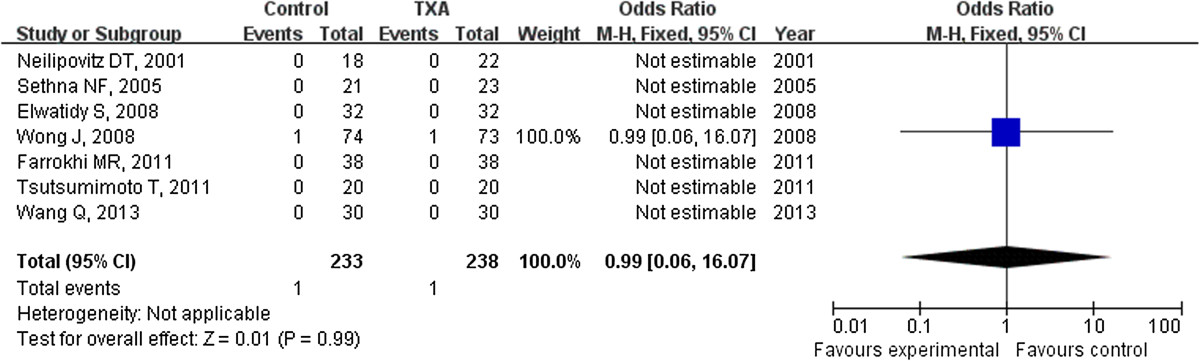
**One patient (1/73) developed an asymptomatic non-Q myocardial infarction in the TXA group and one patient (1/74) had a DVT in the placebo group, it seems insignificant difference for the thrombolic complications between the TXA and placebo group.** TXA = tranexamic acid, SD = standard deviation, IV = inverse variance, and df = degrees of freedom.

Only one study reported the outcome of thrombotic complications, and yielded a null association between thrombosis complications and the use of TXA with ann OR of 0.99 (95% CI: 0.06 to 16.07) (Figure 6).

### Postoperative partial thromboplastic time

**Figure 7:**
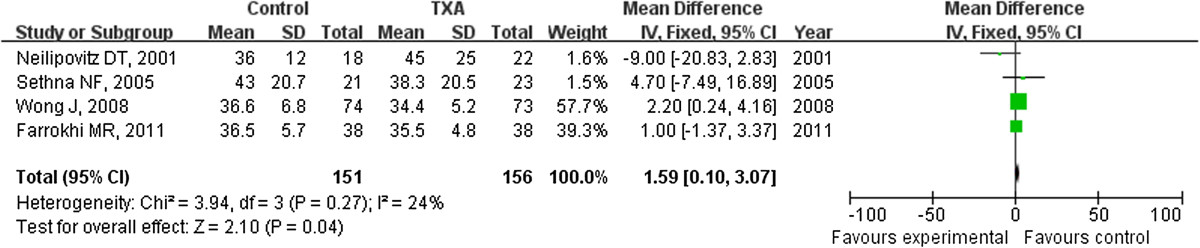
**The postoperative partial thromboplastin time was significantly less in the TXA group compared with the placebo group (weighted mean difference, -1.59 [95% CI, -3.07 to -0.10]; p = 0.04).** TXA = tranexamic acid, SD = standard deviation, IV = inverse variance, and df = degrees of freedom.

**Figure 8:**
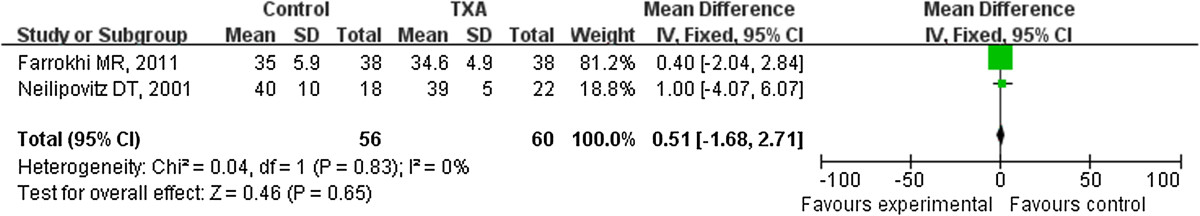
**The outcome of partial thromboplastin time at 24 h postoperative was similar between the TXA group and the placebo group (weighted mean difference, -0.51 [95% CI, -2.71 to 1.68]; p = 0.65).** TXA = tranexamic acid, SD = standard deviation, IV = inverse variance, and df = degrees of freedom.

The outcome measure of PTT at the end of surgery was available in four studies. The postoperative PTT was significantly shorter in the TXA group compared with the placebo group (weighted MD = -1.59 [95% CI, -3.07 to -0.10]; p = 0.04) (Figure 7), however, the average values of PTT in the four studies were vary from 34.4 to 45, all of them were in the normal range (Figure 7). In addition, the outcome of PTT at 24 h postoperative was available in two studies. There was no significant difference for postoperative PTT between the TXA group and the placebo group with weighted MD of -0.51 (95% CI, -2.71 to 1.68), p = 0.65) (Figure 8).

## Discussion

This meta-analysis identified 6 RCTs that compared TXA and placebo intravenous administration in spinal surgery, and showed that the intravenous use of TXA in spinal surgery can significantly reduce the total blood loss, the amount of blood transfusion, and the number of patients needing transfusion; none of the patients in the TXA-treated group had DVT or myocardial infraction, although the PTT was shorter at the end of surgery in the TXA group. These results are similar to the meta-analysis about TXA used in total knee arthroplasty and coronary artery bypass graft surgery [7, 21].

Gill’s meta-analysis [22] regarding the use of antifibrinolytic agents in spine surgery also demonstrated that antifibrinolytic drugs could reduce the amount of blood loss and blood transfusion in spinal surgery, however, the included literatures were not all RCT studies, and thrombotic complications were not considered. Our meta-analysis revealed that the use of TXA in patients who was performed spinal surgery can also reduce the total blood loss. These results are similar to previous clinical trials [23, 24]. However, because the significant heterogeneity existed within included studies, our findings should be interpreted with cautions. We hypothesized that surgical methods, surgical approach, and TXA dose might mainly explained such a heterogeneity, and was confirmed by our subgroup analysis. For surgical methods, the patients who received scoliosis correction were teenagers, they were younger, less weight, and had more surgery involved levels and larger wound area. The number of level fused ranged from 7 to 18 in Neilipovitz’s study [15], some patients accepted anterior soft tissue release and posterior spinal instrumentation concurrently in Sethna’s study [16]. In contrast, there were 2 to 6 vertebrae involved in the surgery procedure. Indeed, heterogeneity was reduced although did not eliminate completely after subgroup analysis (Scoliosis subgroup, I^2^ = 24%, P = 0.25; Non scoliosis subgroup, I^2^ = 48%, P = 0.13). Similar results were obtained from the subgroup analysis by surgical approach and TXA dose. Different anatomical structures will be dissected and injured in different surgical approach, accompanied with different blood loss. Eighteen patients accepted multilevel anterior cervical discectomy, twelve patients accepted laminectomy and discectomy, and others accepted laminectomy in Eiwatidy’s study [19], eight patients accepted lateral thoracotomy or a thoracoscopic procedure and posterior instrumentation concurrently in Sethna’s study [16]. TXA dose may be another resource of heterogeneity, It was difficult to distinguish due to the high dose TXA was administered in the two studies. Moreover, operating time was also known as an important factor influencing the blood loss, it ranged from 89 min to 420 min averagely in these 6 RCTs, according to the experience, longer operating time with more blood loss, anything could influence the blood loss by changing the operating time. Spinal surgery procedures are complex and diversity, blood loss could be effected by many factors. However, the blood loss was significantly decreased in each subgroup with TXA administered. TXA may benefit blood loss reduction in spinal surgery from the exiting data, more powerful evidence was needed in the future.

Our meta-analysis also manifested that the use of TXA significantly reduced the amount of blood transfusions and the rate of patients requiring transfusion when compared with the placebo group. The number of patients requiring transfusion and the amount of blood transfusion were influenced by many factors, such as the amount of blood loss and the indication of transfusion. Blood transfusion was undertaken when the hemoglobin was less than 10 g/L in Farrokhi’s study [18], much higher than the others. We speculated the difference of transfusion threshold may be the reason of heterogeneity, the postulation were supported because the value of I^2^ decreased to 0 when excluded the study. According to the final statistic outcome, the amount of blood transfusion decreased with use of TXA for patients undergoing spinal surgery and fewer patients required blood transfusion. Of these six studies, the amount of blood loss decreased to 25-49% [17, 19], the amount of blood transfusion decreased to 28-80% [15, 19]. The risk of related complication such as immunologic reactions and viral transmission might reduce followed the decrease of blood transfusion.

The safety of use antifibrinolytic agents in spine surgery perioperative has been the focus of the spine surgeon [25, 26]. More data may be needed to access the safety of TXA used in spine surgery because there were few studies about the issue. A meta-analysis about the TXA used in cardiac surgery showed that the complication such as stroke and myocardial infraction did not increase in the TXA group [27]. In our meta-analysis, the thrombotic complication was reported only in Wong’s study, one patient (1/73, 1.37%) developed an asymptomatic non-Q myocardial infarction in the TXA group and one patient (1/74, 1.35%) had a DVT in the placebo group. It seems insignificant difference for the thrombolic complications between the TXA and placebo group. However, the numbers of patients in both groups were small, and larger numbers of patients in samples of randomized controlled trials are needed to support the results. More important, patients were assessed daily for any DVT in 2 studies [15, 18], no special investigations for DVT in other studies. More studies specifically screened for thromboembolism may offer more confidence for TXA administered in spinal surgery. Available data were too limited for any conclusions regarding safety.

TXA inhibits fibrinolysis and consequently stabilizes the fibrin clots [28]. The PTT may be affected by the TXA because of its affect the fibrinolysis system. Some authors [19] found no significant difference in PTT between the TXA and the placebo group. In our meta-analysis, the PTT was significantly less in the TXA group at the end of surgery, while similar between the two groups at 24 h postoperatively. However, the average values of PTT in the four studies were varies from 34.4 to 45, all of them were in the normal range, there was statistically significant but clinically small difference. We cannot conclude that PTT had been affected by the TXA. Further studies are needed to evaluate the change of PTT after TXA used.

There are some potential limitations in our meta-analysis. Because we only selected literature published in English, and searched through MEDLINE, PubMed, EMBASE, and the Cochrane Database, articles written in other languages or included in other database may have been missed, thus the publication bias could not be ruled out. The second limitation was that the difference existed among the methods of spine surgery, which may lead to the heterogeneity and affect the reliability of our study.

## Conclusions

In conclusion, the use of TXA in patients undergoing spinal surgery appears to be effective in reducing the amount of blood loss, the volume of blood transfusion and the transfusion rate. However, data were too limited to reach any conclusions regarding safety of intravenous use of TXA. More high-quality randomized controlled trials are required before applying the administered of TXA in spinal surgery to support the conclusion.

## Authors’ original submitted files for images

Below are the links to the authors’ original submitted files for images. Authors’ original file for figure 1 Authors’ original file for figure 2 Authors’ original file for figure 3 Authors’ original file for figure 4 Authors’ original file for figure 5 Authors’ original file for figure 6 Authors’ original file for figure 7 Authors’ original file for figure 8
