# Supplementary figures and images for: Effectiveness of tranexamic acid in reducing blood loss in spinal surgery: a meta-analysis
Source: BMC Musculoskelet Disord. 2014 Dec 22;15:448. doi: 10.1186/1471-2474-15-448 (PMC4326491; doi:10.1186/1471-2474-15-448)

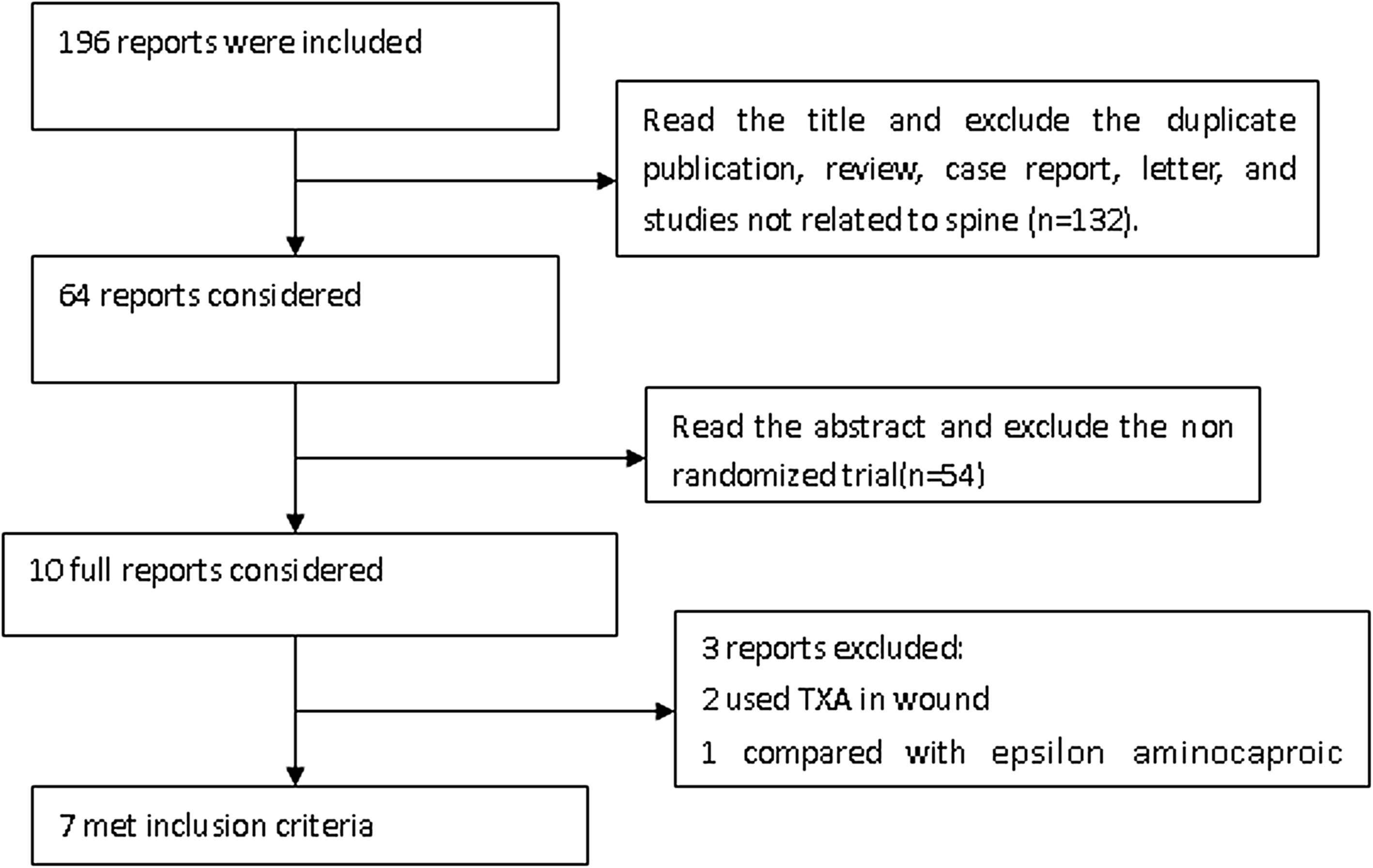

Supplement: Supplementary file 1 — Authors’ original file for figure 1 [file 12891_2014_2399_MOESM1_ESM.tif]

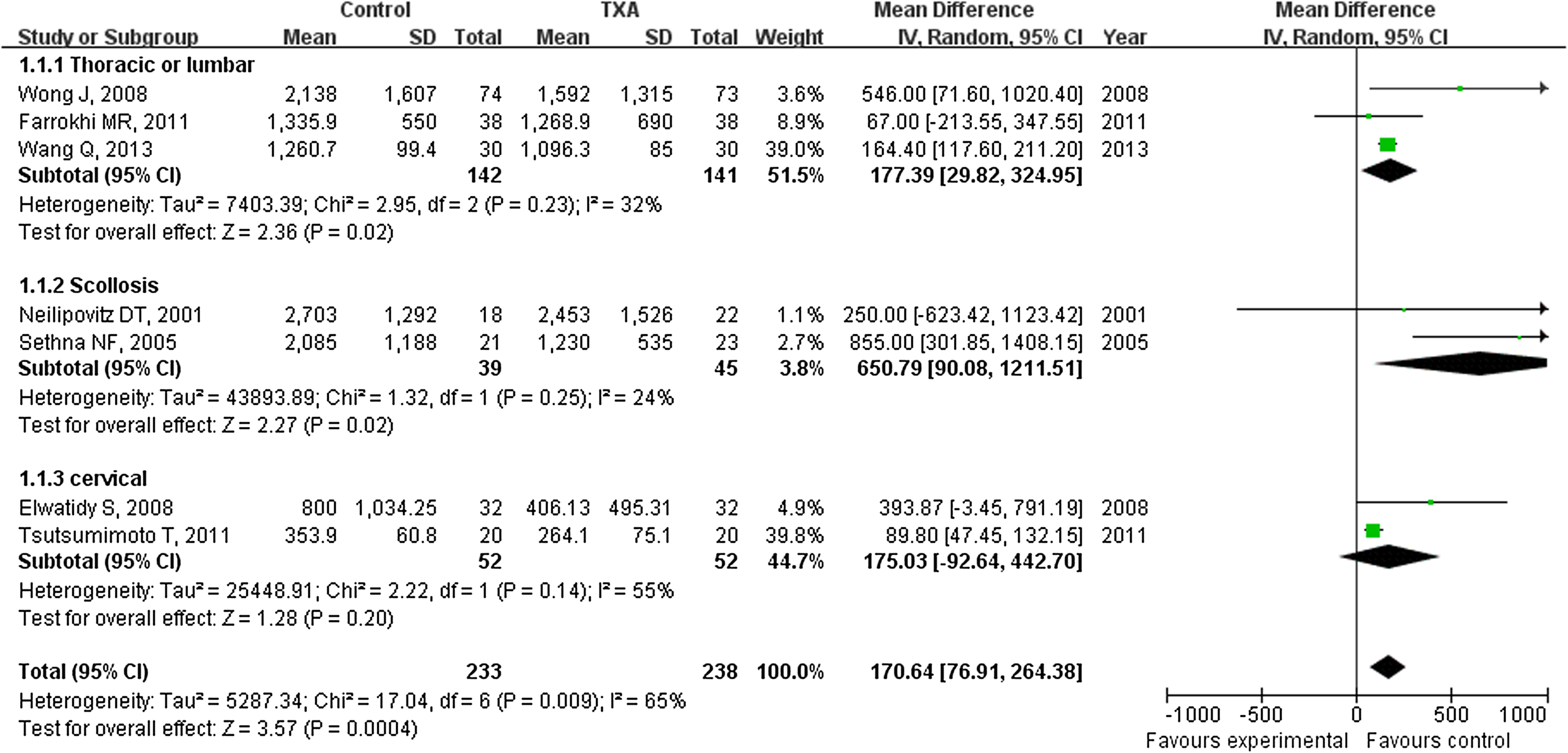

Supplement: Supplementary file 2 — Authors’ original file for figure 2 [file 12891_2014_2399_MOESM2_ESM.tif]

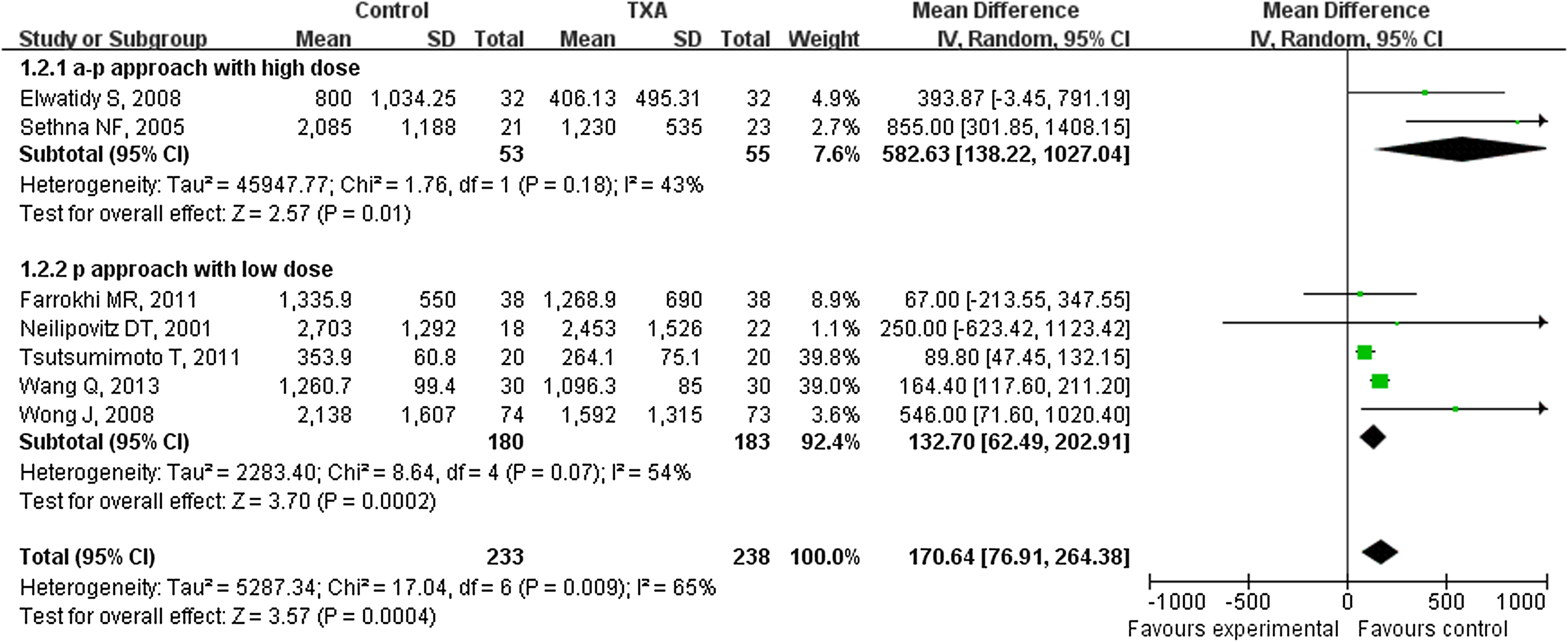

Supplement: Supplementary file 3 — Authors’ original file for figure 3 [file 12891_2014_2399_MOESM3_ESM.tif]

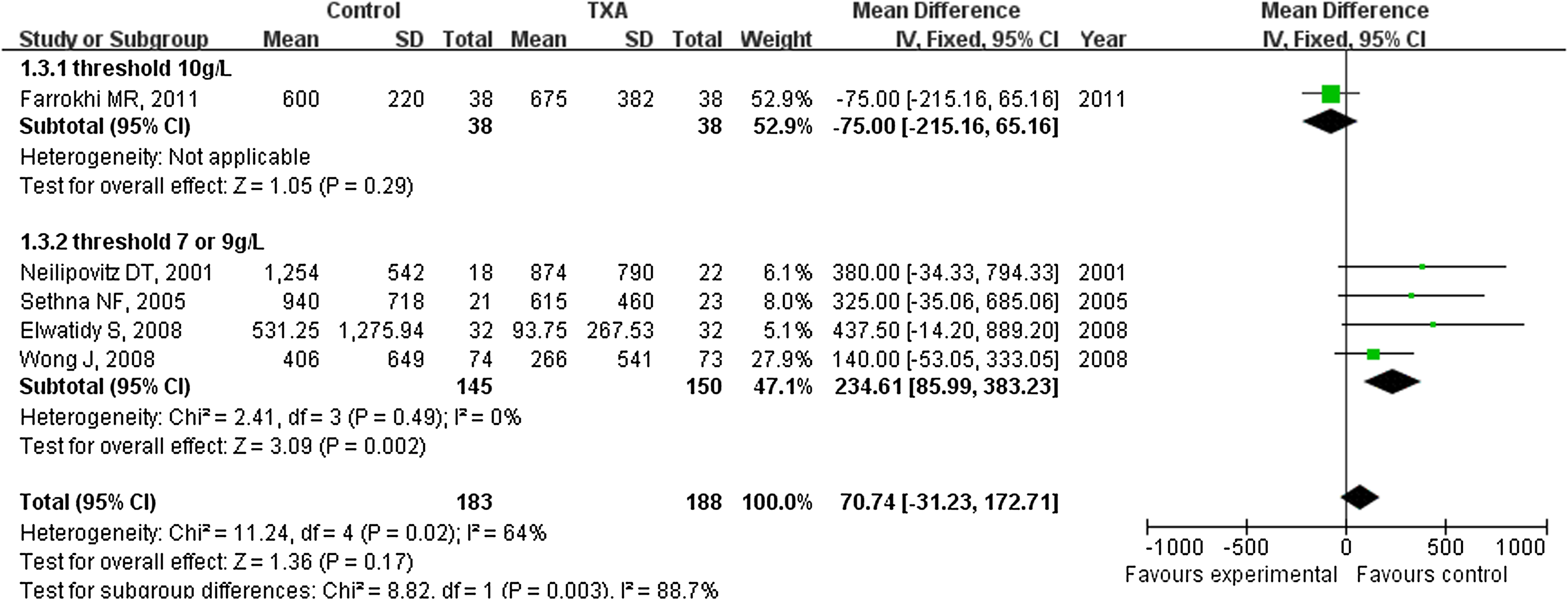

Supplement: Supplementary file 4 — Authors’ original file for figure 4 [file 12891_2014_2399_MOESM4_ESM.tif]

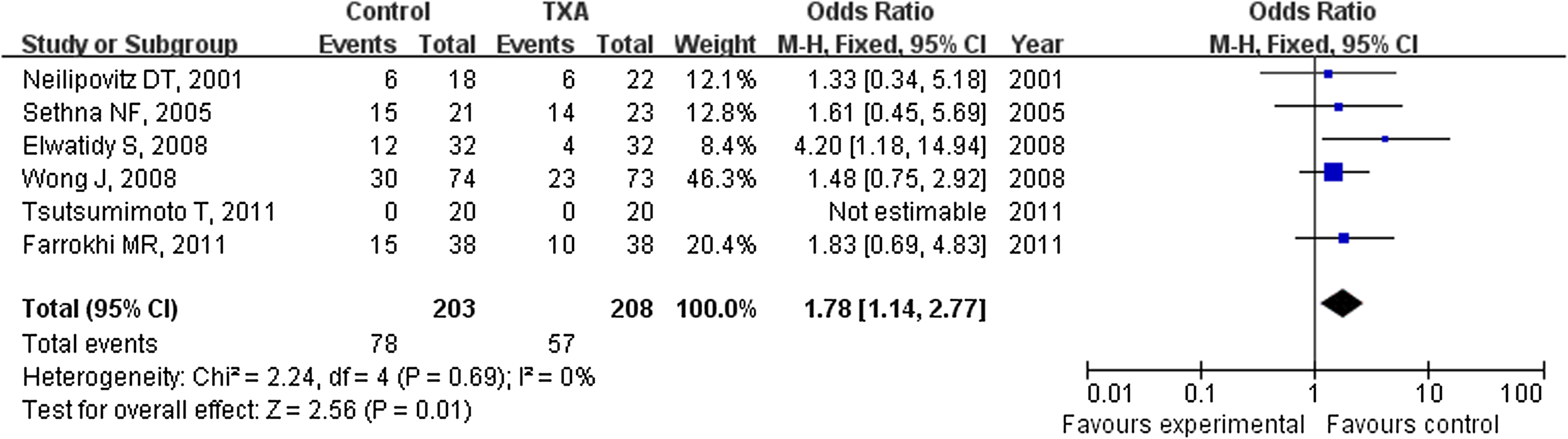

Supplement: Supplementary file 5 — Authors’ original file for figure 5 [file 12891_2014_2399_MOESM5_ESM.tif]

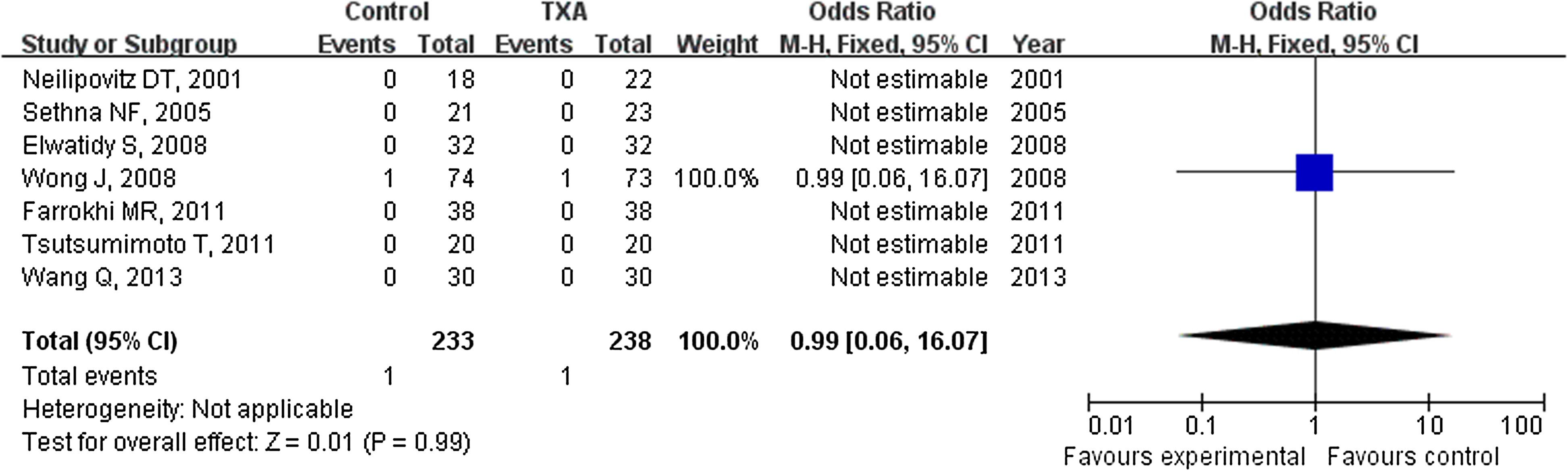

Supplement: Supplementary file 6 — Authors’ original file for figure 6 [file 12891_2014_2399_MOESM6_ESM.tif]

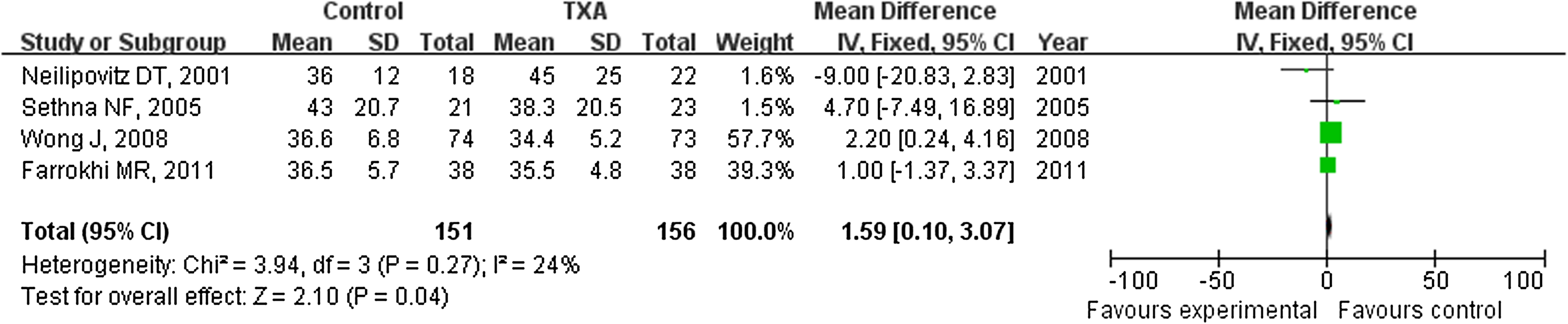

Supplement: Supplementary file 7 — Authors’ original file for figure 7 [file 12891_2014_2399_MOESM7_ESM.tif]

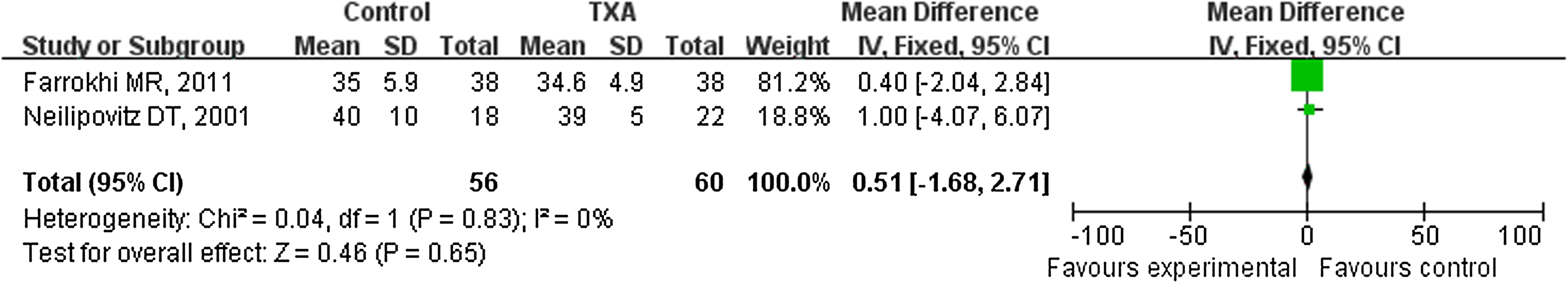

Supplement: Supplementary file 8 — Authors’ original file for figure 8 [file 12891_2014_2399_MOESM8_ESM.tif]
